# Leveraging Twitter to gauge evacuation compliance: Spatiotemporal analysis of Hurricane Matthew

**DOI:** 10.1371/journal.pone.0181701

**Published:** 2017-07-28

**Authors:** Yago Martín, Zhenlong Li, Susan L. Cutter

**Affiliations:** Department of Geography and Hazards and Vulnerability Research Institute, University of South Carolina, Columbia, South Carolina, United States of America; Purdue University, UNITED STATES

## Abstract

Hurricane Matthew was the deadliest Atlantic storm since Katrina in 2005 and prompted one of the largest recent hurricane evacuations along the Southeastern coast of the United States. The storm and its projected landfall triggered a massive social media reaction. Using Twitter data, this paper examines the spatiotemporal variability in social media response and develops a novel approach to leverage geotagged tweets to assess the evacuation responses of residents. The approach involves the retrieval of tweets from the Twitter Stream, the creation and filtering of different datasets, and the statistical and spatial processing and treatment to extract, plot and map the results. As expected, peak Twitter response was reached during the pre-impact and preparedness phase, and decreased abruptly after the passage of the storm. A comparison between two time periods—pre-evacuation (October 2^th^-4^th^) and post-evacuation (October 7^th^-9^th^)—indicates that 54% of Twitter users moved away from the coast to a safer location, with observed differences by state on the timing of the evacuation. A specific sub-state analysis of South Carolina illustrated overall compliance with evacuation orders and detailed information on the timing of departure from the coast as well as the destination location. These findings advance the use of big data and citizen-as-sensor approaches for public safety issues, providing an effective and near real-time alternative for measuring compliance with evacuation orders.

## 1. Introduction

Tropical cyclones represent one of the costliest and devastating threats for populations in both the developed and developing world, with long-lasting consequences that extend over several years [1, 2]. The overall profile for natural hazard losses in the U.S. since 1960 finds that tropical cyclones represent roughly 26% of the total losses [3], but this percentage has been increasing since 2000 and as of 2015 represents 41% of the total loss from natural hazards [4]. While the projections on frequency and intensity of tropical cyclones remain inconclusive [5], there is increasing risk exposure as population and assets continue to shift to coastal areas [6].

While among the costliest hazards, the death toll from tropical cyclones in the U.S. is relatively low, less than 5% of the hazard fatalities. This is largely due to the protective actions adopted by coastal populations: sheltering in place and evacuation. During tropical cyclones, evacuations are primarily ordered for those who live on the coast or in adjacent low-lying areas due to the anticipated storm surge [7]. However, the compliance rates with evacuation orders are often significantly less than 100%, with many coastal residents preferring to ride out the storm rather than leave. The decisions to stay or go depend on several factors such as the perception of risk, prior experience, and the severity of the storm itself [8–11]. Traditionally, evacuation rates were determined by post-evacuation questionnaire surveys months following the hurricane [12–15]. Traffic counts have also been used, but these routinely underestimate the number of people evacuating [16] because the average number of occupants per car is rarely known or estimated. One of the challenges then of emergency management is to assess evacuation order compliance and movements in a timely fashion not only to get people out of harm’s way faster, but to facilitate the re-entry into the evacuation zone as quickly as possible once the storm has passed.

Extreme events have the power to attract the public’s attention and prompt protective actions. Social media has changed the way we receive information and how we communicate [17]. Social media platforms such as Twitter and Facebook offer real-time information and round-the-clock situational awareness and provide a two-way communication channel increasingly used by authorities, businesses, and researchers to quickly grasp public opinion and activity. Today’s social media is available on mobile technologies, frequently with built-in Global Positioning System (GPS) receivers. The availability of location-based social networks with geo-tagged social media data has noticeably improved emergency management research and practice [18]. In fact, emergency and disaster management accounts for 27% of the Twitter data topical applications in reviewed papers [19]. Among the many current social media platforms, Twitter is the most often used data source in studies about emergency situations [20–22]. Twitter enables the user to create, share, read and reproduce information in a simple and fast manner by limiting the content to 140 characters. These features attract 313 million users per month [23], producing more than 7,000 tweets per second [24].

This paper examines the exchange of hazard information triggered by the most powerful Atlantic Basin hurricane of the 2016 season, Hurricane Matthew. Twitter data are used to gauge the social media reaction to the disaster and the protective actions taken by residents from both a spatial and temporal perspective. More significantly, the paper proposes an approach to leverage social media content to measure the evacuation response in terms of evacuation timing, re-entry, and destinations for safety. Two themes provide the focus for this paper. First, the paper explores the spatiotemporal variability in social media (Twitter) information exchange before, during, and after Hurricane Matthew and whether such differences are due to proximity to the areas of potential impact. The second theme takes a more granular look at South Carolina tweets and examines specific responses to mandatory evacuation orders in South Carolina regarding the number of evacuees, the timing of departure and re-entry, and geographic destination.

## 2. Social media usage in disasters

Most of the social media usage in a disaster context is focused in the preparedness and response phases of the emergency management cycle. This is to be expected given the real-time need for situational awareness by the public and emergency managers. The concept *crisis informatics* has been used in the literature to refer to both data and information about emergency response from both official responders and the public. For example, Palen et al. [25] and Ukkusuri et al. [26] have helped to further understanding today’s crisis informatics, including the use of digital networks (social media) as a mechanism to gauge civil response. In the context of natural hazards, temporal patterns have been identified based on the emergency management cycle (preparedness, response, recovery, mitigation) as well as the affected region or place, the type of event, and characteristics of the event. For instance, the highest volume of social media content in the Haiyan typhoon was produced days after the landfall [27], illustrating the response/recovery phase. This same pattern was found on another Philippine typhoon, Yolanda in 2013, where the social media activity peak was reached during the response phase, several hours after of the landfall [28]. In an earthquake example, Crooks et al. [29] and Sakaki et al. [30] leveraged social media response to quickly detect the time and location of the earthquake, essentially using humans as sensors. In a broader study, Huang and Xiao [31] mined Twitter content to assess the reaction during different disaster phases of Hurricane Sandy, concluding that the activity reached its peak during the initial impact and subsequent hours. This finding was also confirmed by Murthy and Gross [32] and Kryvasheyeu et al. [33, 34], who found peak Twitter content on the day of the landfall. Hurricane Irene was also analyzed, finding similar temporal results with the highest number of tweets correlating with the peak of the event [21].

Research using geotagged social media data and identifying spatial patterns of social media users is widespread [35–37]. Within disaster management, examples of spatial social media content are found in earthquake, wildfire, tropical cyclone, or flood events [20, 38–40]. Studies have shown people physically close to a disaster event tend to engage more in disaster-related content in social media. Further, several authors have discussed that when power or phone lines are down or collapsed, Twitter remained active [41, 42]. Graham et al. [43] found through visual interpretation that the highest disaster-related Twitter activity was, as expected, near flooded areas in the United Kingdom during the major floods of 2012. Introducing the distance to the actual floods, Herfort et al. [44] and de Albuquerque et al. [22] proved the same connection between Twitter content and the proximity to a disaster. In the case of tropical cyclones, Kryvasheyeu et al. [33] found a sharp decline in Sandy-related Twitter activity as the distance between the most populated cities in the country and the path of the hurricane increased. In a more focused study on the spatial distribution of Tweets from Hurricane Sandy, Shelton et al. [39] concluded that the physical distance from the city had no significant relationship with the social media activity about Sandy on Twitter.

Researchers have also been interested in looking at human mobility and social interaction patterns using geotagged social media data. For example, Hasan et al. [45] utilize geotagged tweets to identify the spatiotemporal patterns of aggregated and individual mobility in a city. Sadri et al. [46] develop a modeling approach to explore social interaction networks based on social media interactions. Particularly relevant for our study in the disaster context are the contributions made by Wang and Taylor [47, 48], who disclosed spatiotemporal patterns of human mobility in different disasters by leveraging geotagged tweets. They concluded that mobility in urban environments is altered during disaster events and this disturbance and its duration depend upon the characteristics and location of the event. Chae et al. [49] offered a tool to visually show spatial patterns of Twitter user distribution before, during, and after Hurricane Sandy and the 2013 Moore (Oklahoma) tornado. Building on this scholarship, Chen et al. [50] theorized about the utility of Volunteering Geographic Information (VGI) such as geotagged tweets in disaster management and in a hypothetical mass evacuation. Our work applies these findings to a case study of an actual hurricane evacuation in order to evaluate the potential for social media to assist in the quantification of evacuation participation and compliance by residents.

Exploiting social media as a new data stream for evacuation participation addresses some of the issues with the existing methods for behavioral data in hurricane evacuation response. For example, the traditional way of assessing evacuation rates involved surveying the affected population dating back to the early 1960s. While there is a rich historic record of these studies [12], most were funded by federal agencies as part of post-event assessments of evacuations plans. Questionnaire surveys are done by mail and phone but the response rates have been declining. The traditional sampling frames based on address and landline phone are more problematic now because of transition away from landlines to mobile phones and an increasing percentage of unlisted landline numbers. Web-based surveys are cheaper, can be deployed faster, but require internet connectivity and more significantly, user familiarity. Additionally, web surveys can exclude important segments of society (elderly, less educated, the poor) as well as residents in small towns and rural areas [51]. Thus the ability to generalize to a broader population (socially and geographically) is becoming more limiting. Response rates for phone surveys are generally higher (27%) than for mail questionnaire (7%), or web-based surveys (2%) based on a direct comparison in Australia [52]. Direct comparisons of web and mailed questionnaire response rates are quite variable, but on average, mail survey response rates are higher by an average of 20% according to a meta-analysis of US surveys [53].

Increasingly, states and researchers are looking to integrate traffic data into evacuation planning [54]. However, such traffic counts are often devoid of any behavioral assumptions about who is evacuating (in terms of demographic characteristics) and how many people are in each vehicle [55]. Thus the count of cars leaving an area may grossly underestimate the number of households or individuals leaving unless calibrated by the number of people per car (or the number of cars per household that would be used in an evacuation). For example, Dow and Cutter [16] found that one-quarter of the evacuees leaving Charleston in response to Hurricane Floyd (1995) took two or more cars, regardless of household size. By analyzing Twitter users we obtained a sufficiently large dataset to establish spatiotemporal patterns of people’s evacuation behaviors without relying on questionnaire surveys or traffic counts.

## 3. Hurricane Matthew and study area

Hurricane Matthew was the strongest storm recorded in the Atlantic during the 2016 season, the first Category 5 hurricane since 2007 in this basin [56]. Matthew formed from a tropical wave pushed off the African coast became a named storm September 28^th^, 2016. Hurricane Matthew rapidly intensified over the warm waters of the Caribbean to a Category 5 storm, and made its first landfall in Haiti as a Category 4 hurricane where its torrential rains caused 546 deaths [56]. On its way north, Matthew made a second landfall in Cuba and a third landfall in the Bahamas causing significant economic damage in both countries. By October 7^th^, Matthew’s track was parallel to the coastline of Florida remaining just offshore as a Category 3 hurricane. A day later, the system started to weaken rapidly as it neared the Georgia coast and made its final landfall (October 8^th^, 1500 UTC) near McClellanville, South Carolina as Category 1. On October 9^th^, while still close to the North Carolina coastline, Matthew was downgraded to an extratropical low, but its windfield and torrential rainfall continued to affect coastal North Carolina and Virginia.

Given the track and early intensity of the storm, coastal residents from Florida to North Carolina were advised to evacuate in advance of the storm. Voluntary and mandatory orders were initially given on October 5^th^ and 6^th^ for the 1.5 million residents living in evacuation zones on the Atlantic coast of Florida. Georgia residents east of Interstate 95 were mandated to evacuate on October 6^th^. The South Carolina mandatory evacuation order was given by the Governor on October 4^th^ and coastal counties began their evacuation the next day. South Carolina implemented lane reversals on the Interstate 26 to speed up the evacuation.

**Fig 1:**
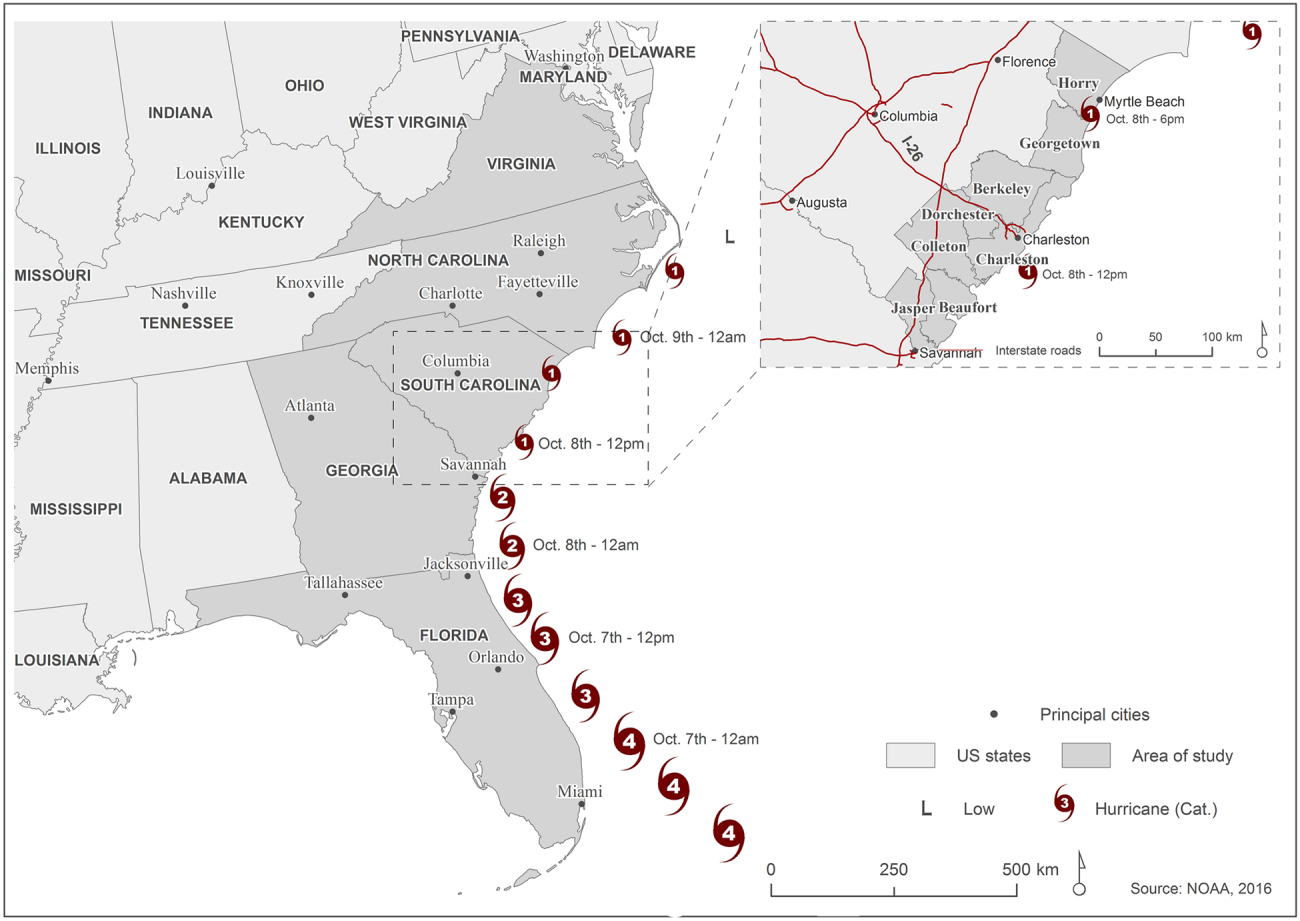
Hurricane Matthew and the study area for the regional analysis (left) and the local analysis (right). The Hurricane Matthew track was obtained from the National Oceanic and Atmospheric Administration (NOAA: http://www.nhc.noaa.gov/gis/archive_besttrack.php).

Fig 1 presents the two different study areas established for the research questions. For the spatiotemporal analysis, the entire area affected by Matthew was considered, including the states of Florida (FL), Georgia (GA), South Carolina (SC), North Carolina (NC), and Virginia (VA). In the evacuation behavior analysis, the focus was on the coastal counties of South Carolina (Jasper, Beaufort, Colleton, Charleston, Dorchester, Berkeley, Georgetown, and Horry).

## 4. Data collection and preprocessing

**Fig 2:**
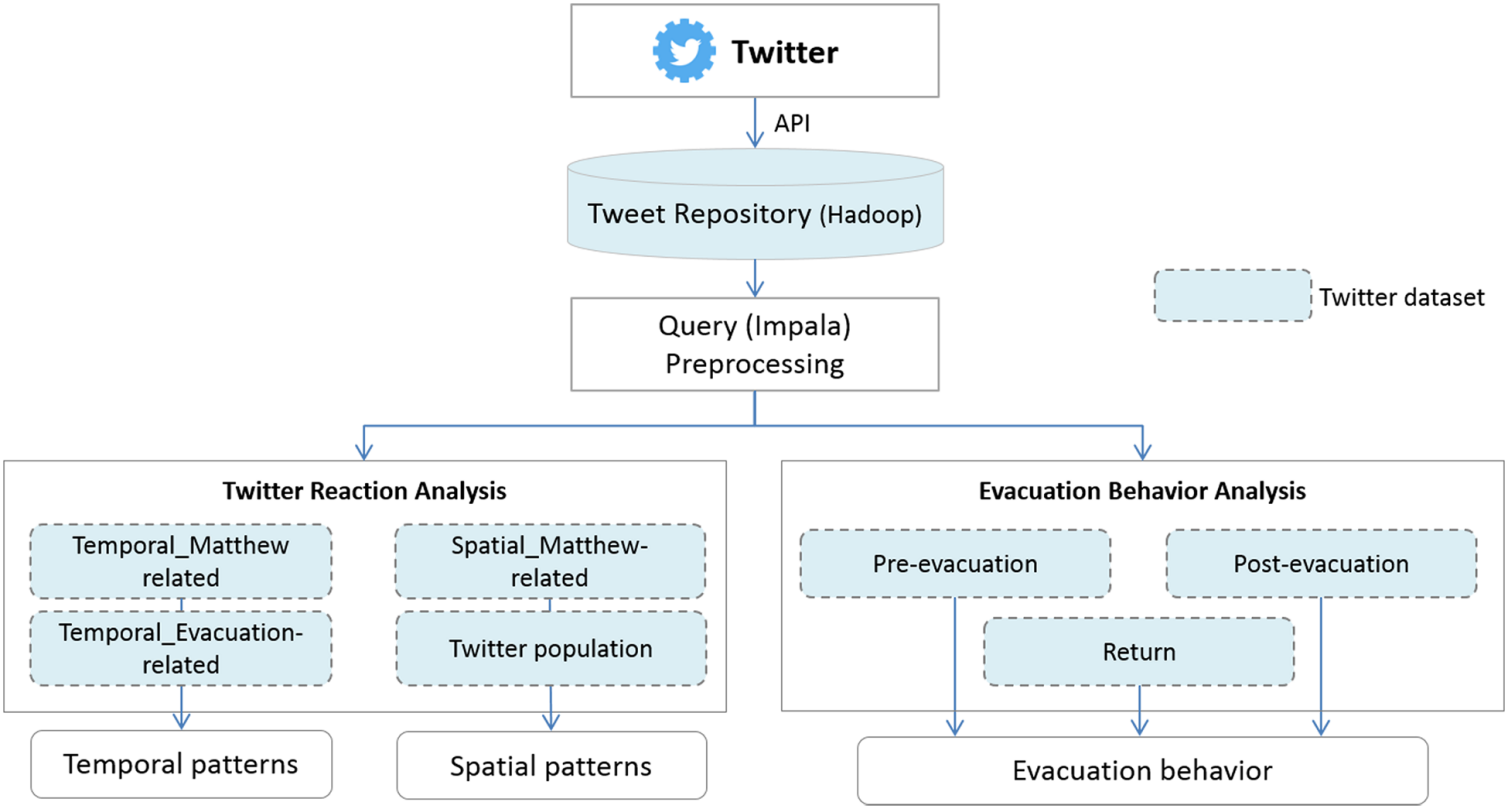
Overview of the research approach.

Our research approach is based on different datasets, study areas, and timeframes (Fig 2). Over 418 million geotagged tweets within the continental U.S. were collected using the Twitter Stream Application Programming Interface (API), comprising a six-month period from July 7^th^, 2016 until December 31^th^, 2016. These tweets are stored and managed in a Hadoop (http://hadoop.apache.org) environment that served as tweet repository for this study. The repository was then queried with Apache Impala (https://impala.incubator.apache.org) using spatiotemporal criteria and keywords in the tweet message and hashtags, obtaining seven Twitter datasets covering different spatial regions, keywords, time periods, and spatial accuracies (Table 1). The locational accuracy of a geotagged tweet depends on how a Twitter user shares his/her location when posting a tweet. The location can be shared in the format of place names (e.g. country, state, city) or the exact latitude and longitude (point-level, determined by the device’s GPS or other signals such as cell tower).

**Table 1:** Twitter datasets.

	Dataset name	Region	Keywords	Time period	Locational accuracy	Number of tweets
Temporal analysis	Temporal Matthew-related	FL, GA, SC, NC, VA	matthew, hurricane*, evac*, storm*	10/02–10/11	State, City, Point	64,189
	Temporal Evacuation-related	FL, GA, SC	evac*	10/02–10/11	State, City, Point	7,735
Spatial analysis	Spatial Matthew-related	FL, GA, SC, NC, VA	matthew, hurricane*, evac*, storm*	10/02–10/11	City, Point	54,509
	Twitter Population	FL, GA, SC, NC, VA	*	09/15–10/15	City, Point	4,706,685
Evacuation behavior	Pre-evacuation	SC coastal counties	*	10/02–10/04	City, Point	13,370
	Post-evacuation	US	*	10/07, 6pm—10/09, 10am	City, Point	13,685
	Return	US	*	10/09, 10am– 10/19	City, Point	19,216

### 4.1 Datasets for spatiotemporal analysis

The *Temporal Matthew-related* dataset contains the tweets (64,189) from the five affected states during the time period Matthew was active in the region (October 2^nd^–October 11^th^, 2016). Four keywords were applied in the query (“matthew”, “hurricane*”, “evac*”, “storm*”) in accordance with other hurricane studies [57, 33]. In this case, the lowest spatial accuracy was kept to the state level. The wildcard “*” was used for fuzzy query to include other variants of the keyword, such as hurricanes and “hurricanematthew” (hashtag).

The *Temporal Evacuation-related* dataset is a subset of the previous dataset composed of 7,735 tweets in the states that ordered voluntary or mandatory evacuations for Matthew (Florida, Georgia, and South Carolina). The timeframe and the spatial accuracy remained unaltered while the keywords of the query were reduced to “evac*”.

The spatial analysis requires a finer degree of accuracy than the temporal analytical set. Therefore, the *Spatial Matthew-related* dataset only includes the tweets with city-level and point-level accuracy. The keywords were kept similar to the temporal analysis. The number of tweets contained in this dataset was 54,509.

To determine the universe of Twitter users in the study area, a dataset (*Twitter Population*) containing 4,706,685 tweets within the time span from September 15th, 2016 to October 15th, 2016 was extracted for the five affected states. The keyword was left blank to collect as many tweets as possible. The spatial accuracy needed matched with the requirements of the analysis (at least in city-level).

### 4.2 Datasets for evacuation behavior analysis

South Carolina was used for the coastal evacuation analysis. Thus, the *Pre-evacuation* dataset identifies the largest possible number of users in the coastal counties prior to the evacuation. Consequently, the keyword of the query was left blank. The time span encompassed from October 2^nd^, 2016 to October 4^th^, 2016, with a spatial accuracy set in the city-level or higher. A total of 13,370 tweets formed the dataset after a manual filtering process to remove non-human (bots) users.

*A Post-evacuation* dataset was extracted for the time in which Hurricane Matthew was affecting South Carolina (October 7^th^ 6pm, 2016 –October 9^th^ 10am, 2016). We assumed that by October 7^th^ at 6pm the evacuation had been completed, taking into consideration the night [16] and travel (rainy) conditions (first effects of Matthew). In a similar manner, we understand the return period had not been initiated by October 9^th^ at 10am considering that the trip would have implied driving at night and in rainy and flood conditions. We used the active users from the *Pre-evacuation* dataset in order to narrow down the query to the users that posted in both periods of time (pre and post evacuation). The keyword was left blank (*) in order to gather as many tweets as possible while the spatial accuracy was set on the city-level or higher, covering the United States. This dataset gathered 13,685 records.

The *Return* dataset was collected to analyze the return timeline of the evacuees. Therefore, it gathers the tweets posted from the end of the *post-evacuation* period until October 19^th^, 2016. Spatial accuracy remains in the city-level and the keyword was left blank. This dataset contained 19,216 tweets throughout the United States.

## 5 Twitter reaction to Hurricane Matthew

### 5.1 Temporal analysis

To determine the temporal trends on Twitter activity related to Hurricane Matthew, we generated six hourly plots of Matthew-related tweets. Daily aggregates were also included in the plots to facilitate the overall interpretation.

**Fig 3:**
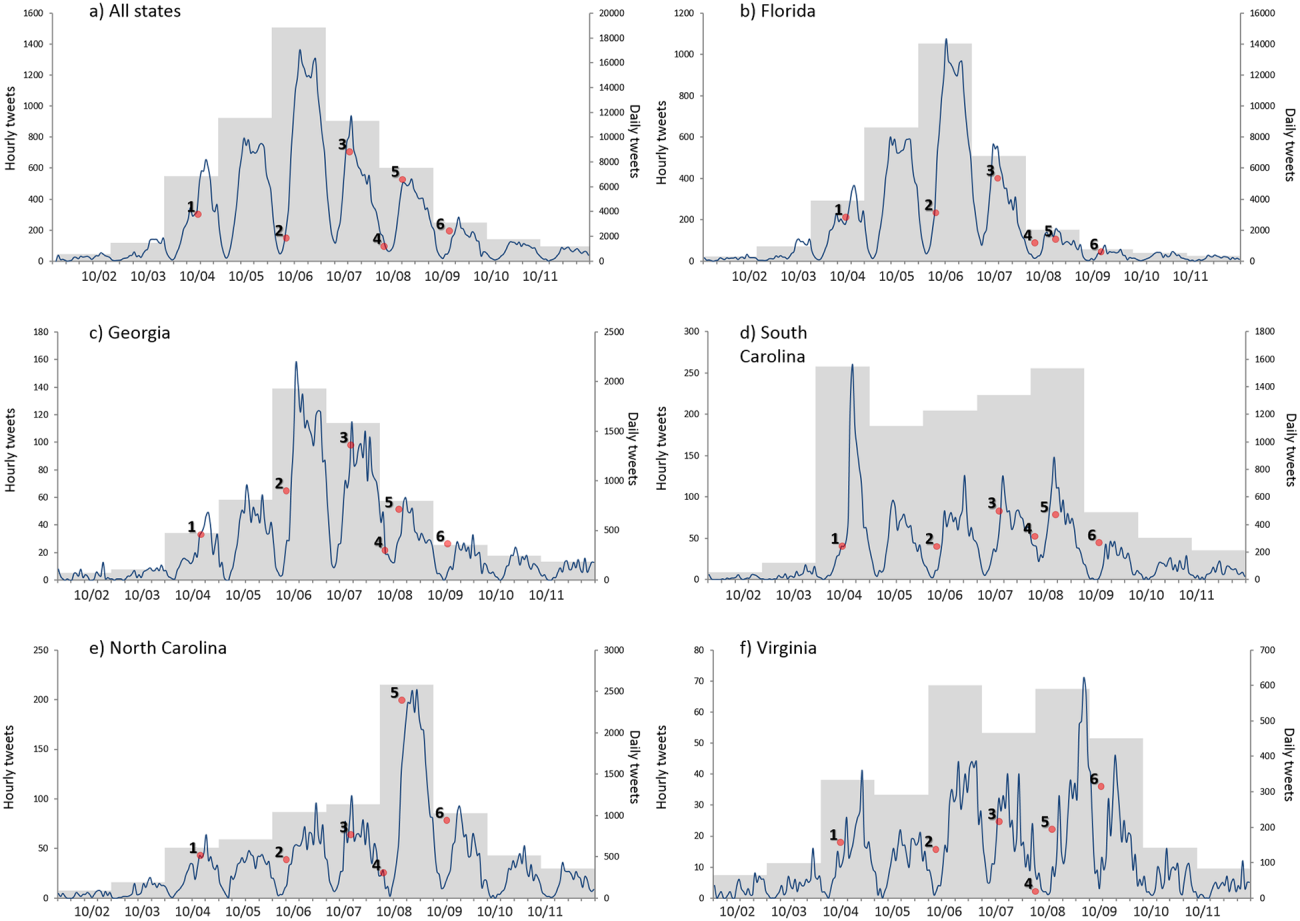
Temporal distribution of Matthew-related tweets for the five states. a) All states b) Florida, 59.7% of the tweets c) Georgia, 10.2% of the tweets d) South Carolina, 12.4% of the tweets e) North Carolina, 12.9% of the tweets f) Virginia, 4.9% of the tweets. Shaded area represents daily tweets, while the hourly tweets are shown on the line graph. Numbered dots indicate the selected key events during the time period: 1. South Carolina evacuation order. 2. Georgia evacuation order. 3. Hurricane eye offshore Daytona Beach (FL). 4. Hurricane eye offshore Savannah (GA). 5. Hurricane eye offshore Charleston (SC). 6. Hurricane eye offshore Wilmington (NC).

In the affected states (Fig 3a), Hurricane Matthew-related tweets started increasing in the afternoon of October 3^rd^ as a consequence of the strength of the hurricane, the impending landfalls in Haiti and Cuba, and the revised forecast of movement in a northern direction with a projected landfall somewhere between Florida and North Carolina. Altogether, the tweeting activity peaked on October 6^th^ and then declined rapidly thereafter. This pattern is highly affected by the timing of the tweets from Florida, as they represent about 60% of the total of tweets in the study region. Regionally, as the forecasted track changed, there was a gradual shift in social media attention between October 6^th^ and October 9^th^. Twitter activity in Florida peaked on October 6^th^ and then declined the following day. Georgia also peaked on October 6^th^, but the drop on October 7^th^ is less noticeable. For North Carolina, the social media attention reached its highest on October 8^th^, the day the hurricane made landfall in South Carolina. Virginia, the northernmost state in the study area, still had a large volume of Twitter activity on October 9^th^ (as the hurricane windfield was still affecting its coast).

South Carolina is an anomaly in Twitter trends. Twitter activity peaked on October 4^th^, dropped the next day, and then gradually reached a second peak on October 8^th^. This pattern reflects two events. The first peak was triggered by the official mandatory evacuation order given by the Governor South Carolina, which caused a blast of attention to the hurricane, as it served as official confirmation of its potential danger. The second peak was caused by the hurricane’s proximity to South Carolina’s coast and impending landfall.

**Fig 4:**
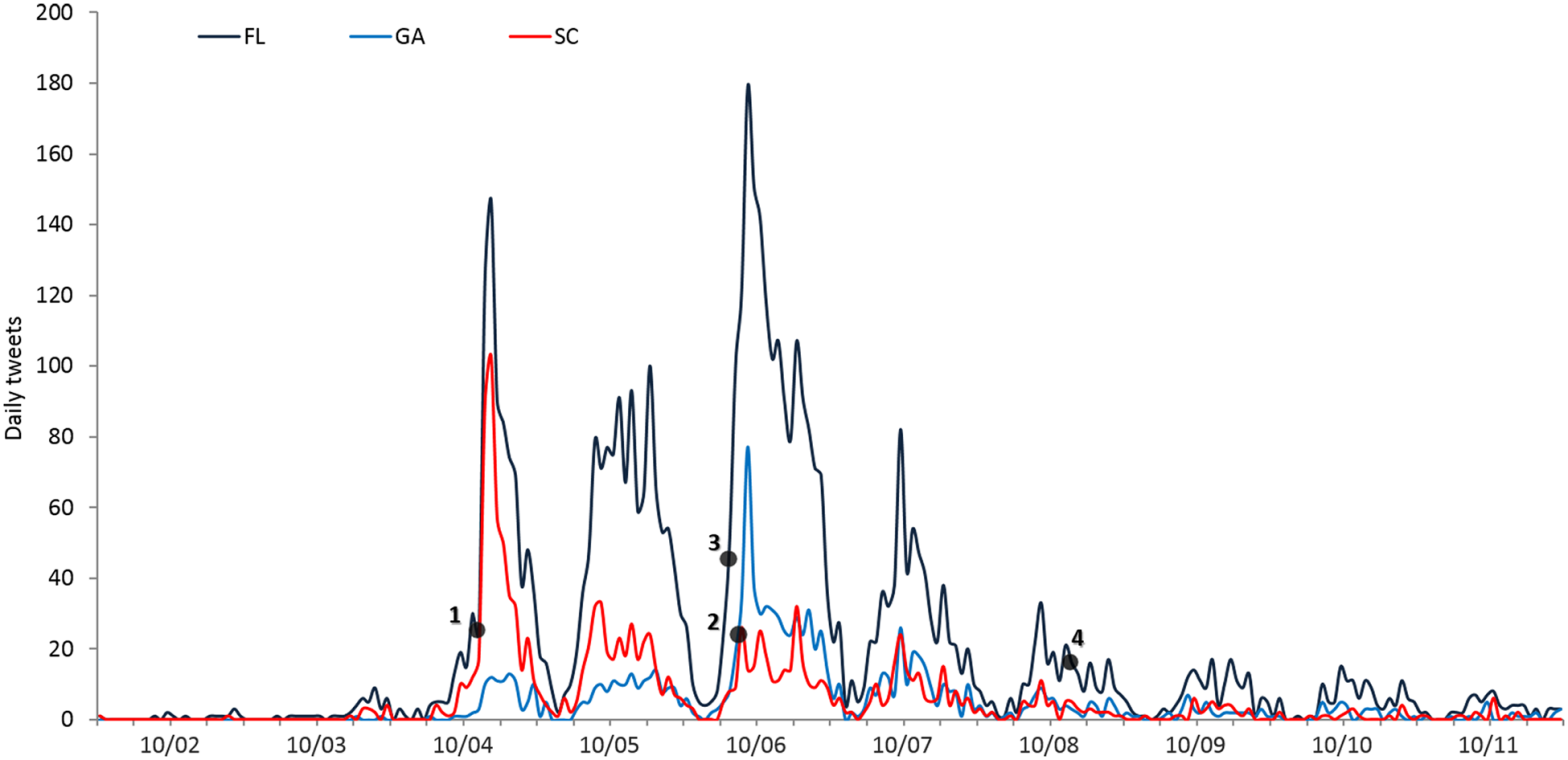
Temporal distribution of evacuation-related tweets. Numbered dots indicate the key events during the time period: 1. South Carolina evacuation order (SC residents in the coastline counties were mandated to evacuate on October 4^th^); 2. Georgia evacuation order (GA residents on the east of Interstate 95 were mandated to evacuate on October 6^th^); 3. Florida evacuation warning by Florida Governor (1.5 million of residents living in evacuation zones on the Atlantic coast of Florida were urged to evacuated on October 6^th^); 4. Landfall near McClellanville, South Carolina (October 8^th^, 1500UTM).

To further investigate Twitter reaction to the evacuation orders, we plotted the evacuation-related tweets for three states that had evacuation orders issued: Florida, Georgia, and South Carolina. As illustrated in Fig 4, evacuation-related tweets showed a different temporal pattern between the states. The South Carolina evacuation order, on October 4^th^ was pre-emptive and heightened Twitter attention in both South Carolina and Florida. Georgia and Florida experienced their peaks on October 6^th^, corresponding to the evacuation orders issued by the two states.

The overall temporal patterns identified in Fig 3 diverges from earlier studies of social media reactions to hurricane threats such as Irene in [21] or Sandy in 2012 [31–33], where the peak of the response was reached when the landfall occurred. Matthew-related content showed a different dynamic, as maximum Twitter activity occurred before the storm’s most intense rainfall and winds were felt in the affected states. In fact, at the time of the landfall, Twitter activity in the study area had dropped considerably. This may have been caused by the unusual track of the storm—an offshore parallel path and the overall weakening of the storm after landfall. Also, this region of the US has more experience with hurricanes and evacuation, resulting in residents paying more attention to preparatory actions in advance of the storm, rather than waiting for landfall (Fig 4). The immediacy of the situational awareness provided by Twitter certainly enhances preparedness in advance and during the storm, which is why it is so widely used by residents and officials [58]. However, there is at least one case study that illustrates the utility of using Twitter during the restoration phase, as was the case with Typhoon Haiyan in 2013 [27].

### 5.2 Spatial analysis

The spatial analysis was carried out at the county level for the whole study area by counting the number of Matthew-related tweets per county with the spatial join function provided by ArcGIS. Following Shelton et al. [39], we normalized the Twitter activity by the Twitter population (active Twitter users from September 15^th^ to October 15^th^ in the study area) to reduce the population bias (Eq 1). The ratio between the number of Matthew-related tweets and the Twitter population accounted for the normalized Matthew-related Twitter Activity (MTA) for each county.

$$MTA=\frac{\text{Matthew}\_\text{related Tweets}}{\text{Twitter Population}}$$ (1)

**Fig 5:**
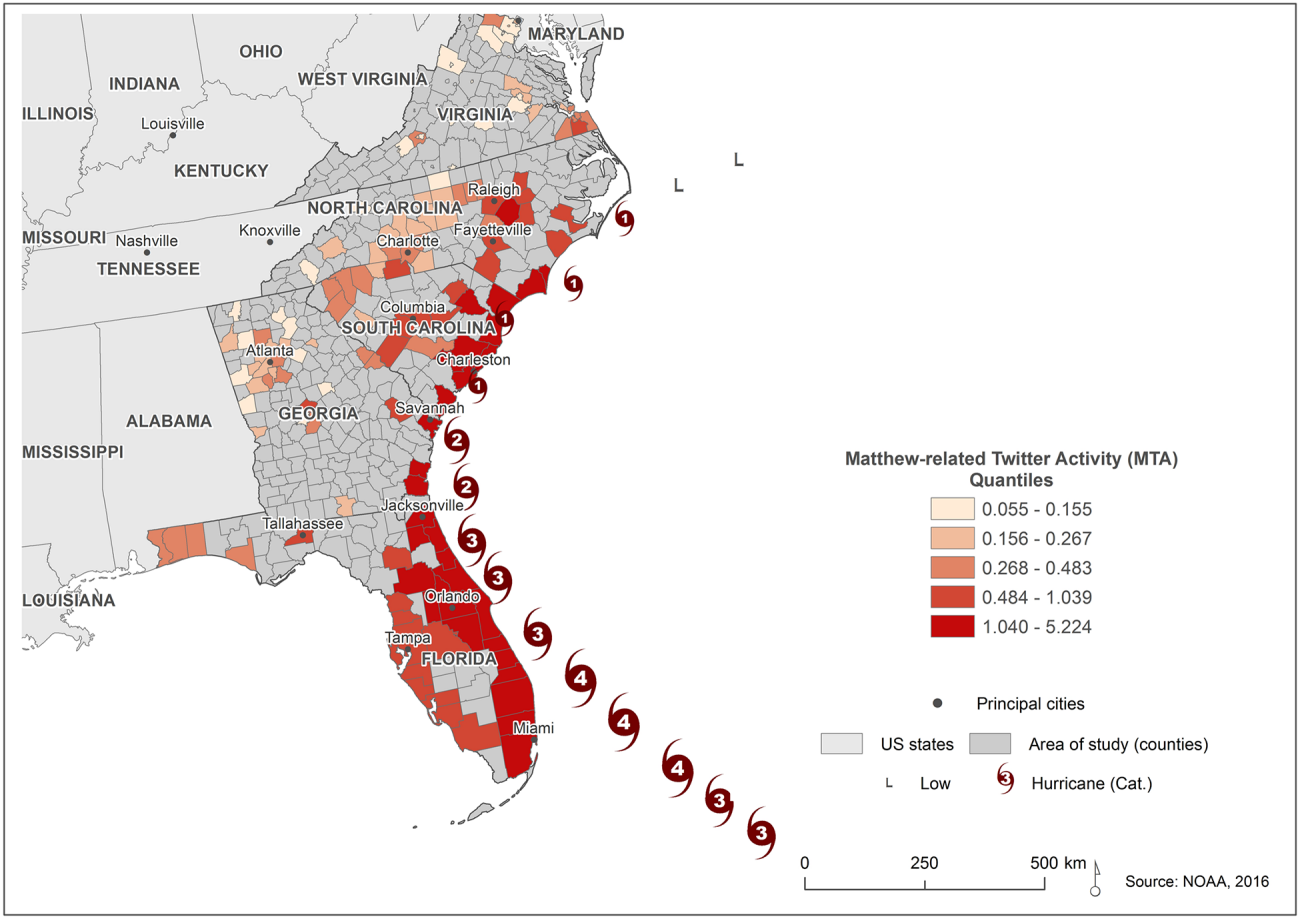
Spatial distribution of Matthew-related Twitter activity in counties with more than 50 active users.

To obtain the active users within the counties of the affected area (Twitter population), we used the *Twitter population* dataset (Table 1). We assigned the location where a user tweeted the most during the time period as their “home” location, using only those users with more than ten tweets in the same city to ascertain that the sample represents the active local users. This threshold of 10 tweets was established based on the distribution of the tweets per user. To avoid outliers in Twitter activity caused by the small amount of users, only those counties with more than 50 active users (151 counties) during that timeframe were used for further analysis. It is worth noting that the subset of 151 counties contains 96.7% of the Matthew-related tweets, as it includes the most populated counties in the study area. Fig 5 shows the spatial distribution of the Matthew-related Twitter activity (MTA) for the subset of counties.

As illustrated in Fig 5, the most intensive Twitter activity was generally recorded along the Atlantic coast of the affected states. Florida exhibits more activity compared to the other four states. This is due to a number of factors: the size of the coastal population in the state, the length of its coastline, the coast-to-coast width of the state where residents on the western coast could be affected, and the decreasing magnitude (category) of the hurricane as it traveled northward. These factors coupled with initial intensification and the uncertainty of the forecasts combined to produce a greater social media response in Florida, even in counties with no Atlantic coastline.

**Fig 6:**
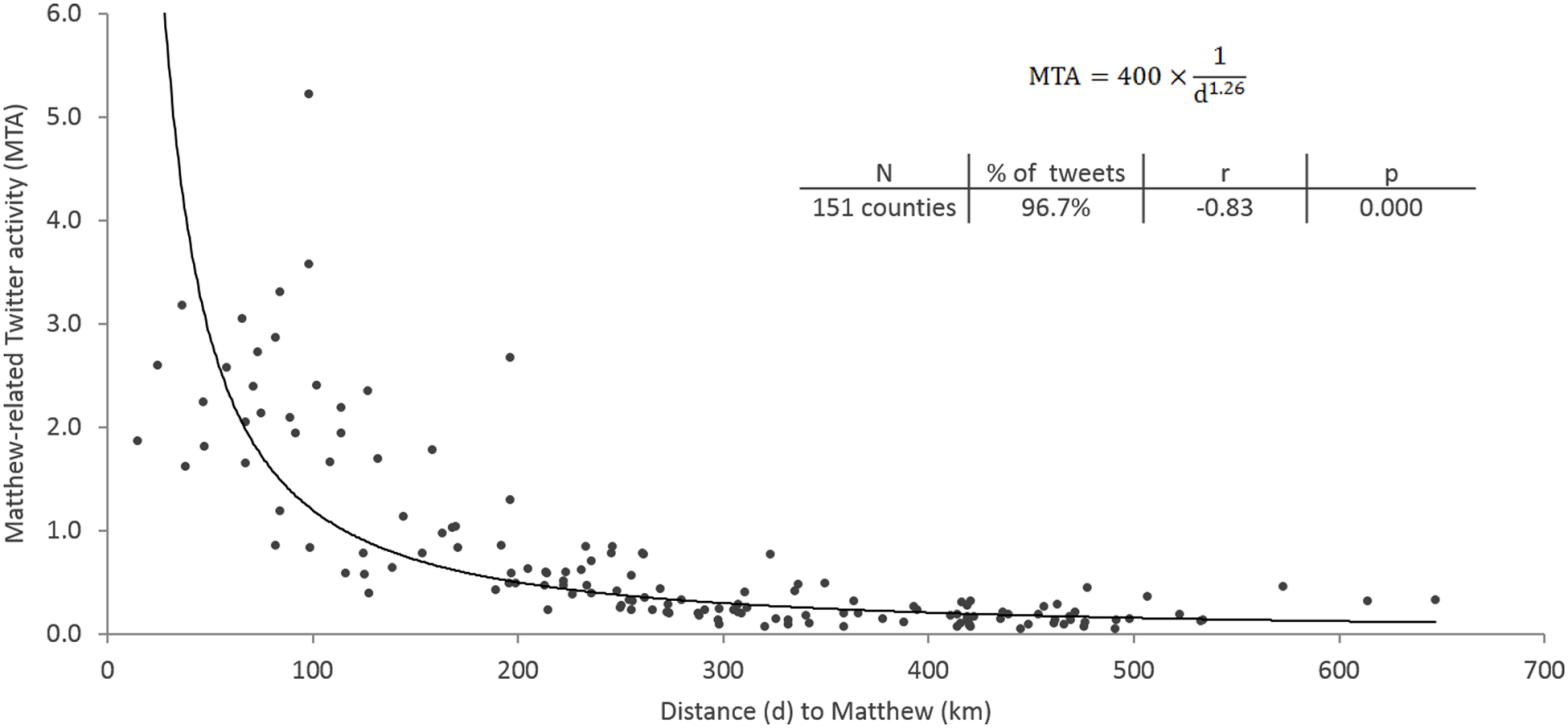
Correlation between Twitter activity per county and distance to Hurricane Matthew.

To further explore the spatial relationship between Twitter activity and Matthew, we conducted a regression analysis between the normalized Twitter activity (*MTA*) per county and the distance from the county centroid to the estimated track of the hurricane (*d*) (sample size: 151). As shown in Fig 6, Twitter activity and the distance to hurricane exhibit significant negative correlation, with r of -0.83. The negative relationship is illustrated by a power best-fit line, showing a sharp decline in the normalized number of tweets as distance increases. This result confirmed the visual pattern of the map (Fig 5). More importantly, the best fit line and its mathematical model (the equation in Fig 6) is consistent with the well-known Distance-Decay function (inverse power law format with the exponent β = 1.26), which describes the effect of distance on spatial and social interactions.

The spatial analysis illustrated that the closer a community is to the threat, the more likely it is to engage in related social media content. This finding confirms and reinforces studies previously conducted for other disaster events [22, 33, 40]. This trend may be driven by the likelihood of personally experiencing the effects of the storm, the individual perception of risk, and the social media discussion about the preparation actions.

## 6 Response to the evacuation order

In the evacuation behavior analysis, we downscaled our study area to three South Carolina Coastal Hurricane Conglomerates used for planning and response actions. These contain eight coastal counties: Horry and Georgetown (Northern Conglomerate); Charleston, Berkeley, and Dorchester (Central Conglomerate); and Beaufort, Colleton, and Jasper (Southern Conglomerate). The focus was on a more detailed analysis of actual evacuation behavior at this local scale—how many Twitter users evacuated, their evacuation destination, and return date.

**Fig 7:**
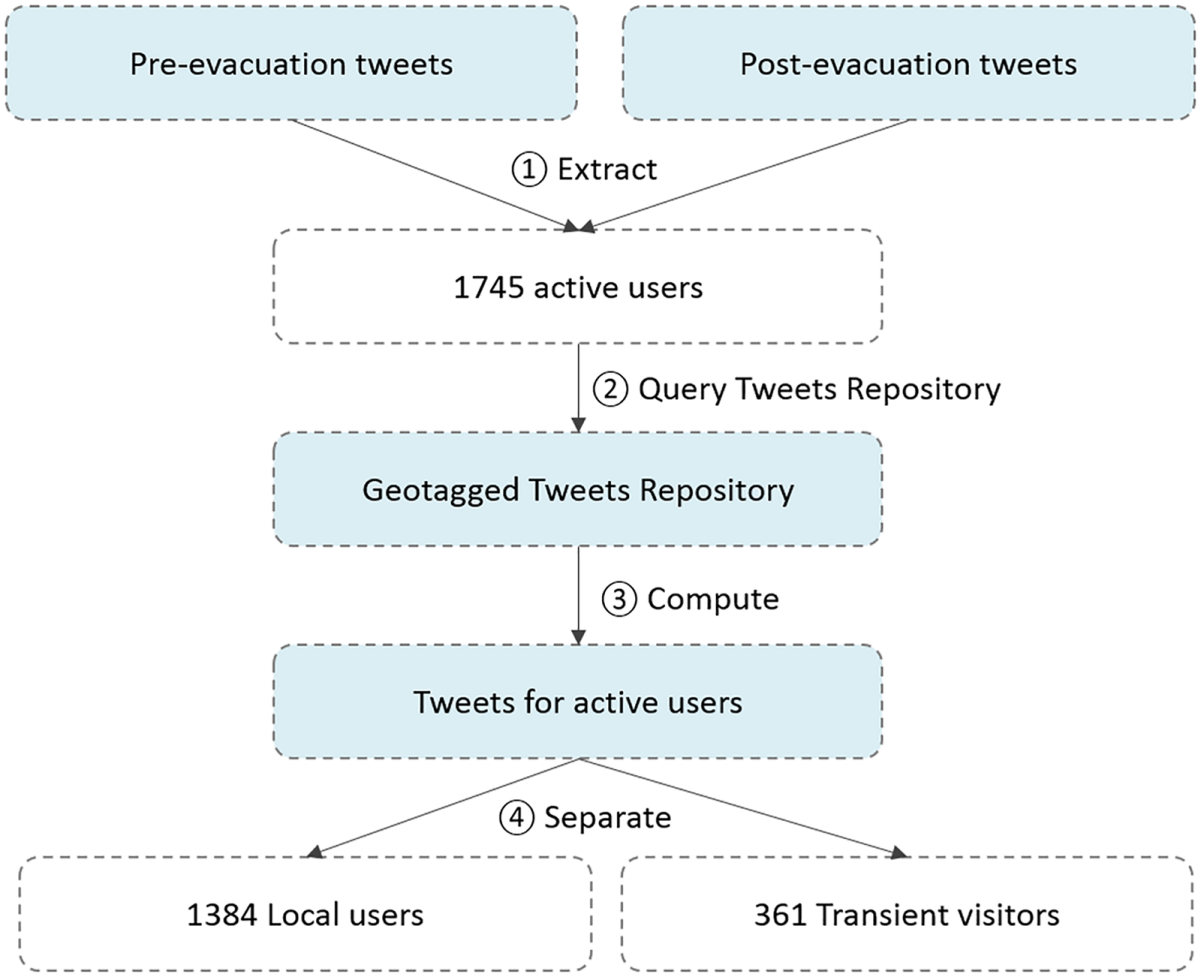
Workflow to obtain the local users.

We first identified the local active Twitter users during the pre-evacuation period (October 2^nd^ to 4^th^) based on the following steps (Fig 7): 1) extraction of the Twitter users who posted at least one message during the pre-evacuation and the post-evacuation timeframe (*pre-evacution* and *post-evacuation* dataset, Table 1); 2) extraction of all tweets (city-level, point-level) posted by the users obtained in the previous step from the *Geotagged Tweets Repository* (Table 1). To limit leisure trips, the national holiday periods (Thanksgiving, November 22–29 and Christmas, December 20–31) were excluded; 3) computation of the median center of the location (coordinates latitude and longitude) of all tweets posted by each user using the median center function provided by ArcGIS. If the location accuracy of a tweet is city-level, the centroid of the city boundary was used as the coordinates for the calculation; and 4) The median centers were then considered as the “home” location for the users, therefore separating the 1384 local users (median center within the coastal counties, 79.3%) from the 361 transient visitors (20.7%). Lastly we calculated the median center of the tweets posted by the 1,384 users during the post-evacuation period (when Matthew was directly affecting South Carolina). This provided a post-evacuation location for each user.

### 6.1 How many users evacuated?

**Fig 8:**
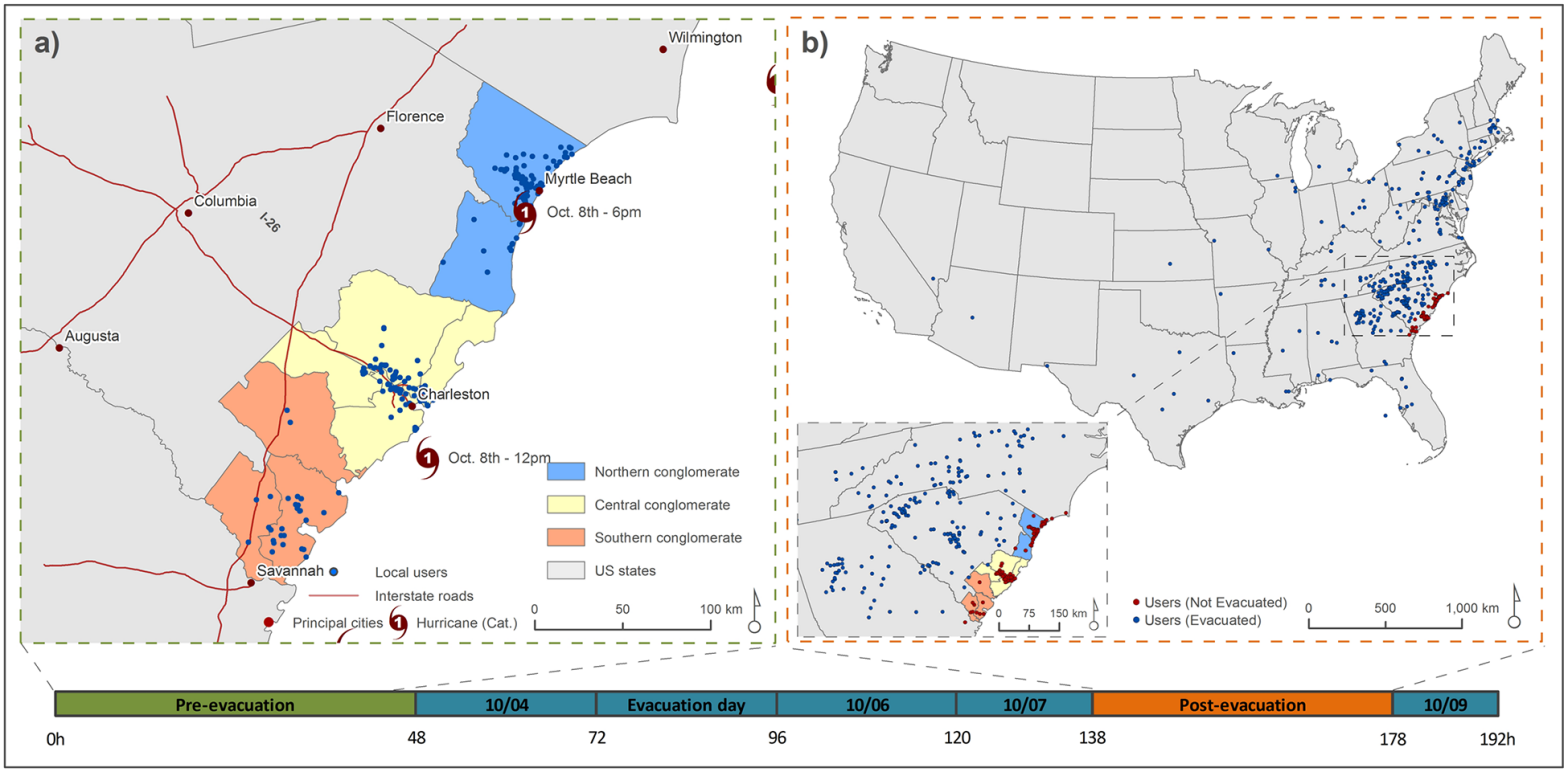
Response to the evacuation order of the 1384 local Twitter users. (a) Local-user locations during pre-evacuation period (10/02-10/04). (b) Local-user locations during post-evacuation period (10/07 6pm– 10/09 10am). Blue dots represent the users who moved outside of the risk area (eight coastal counties), and red dots were the users who did not evacuate.

Fig 8a shows the home location of the 1,384 active local users identified before the evacuation, especially clustering around two major cities: Charleston (Central Conglomerate) and Myrtle Beach (Northern Conglomerate). Fig 8b displays the locations of the same 1,384 users during the post-evacuation period. Blue dots represent the users who moved outside of the eight coastal counties, and red dots show the location of the users who did not evacuate. Table 2 depicts the number of evacuated users in the three conglomerates and the estimated number of evacuees based on the population of each conglomerate. We only considered users as evacuees if they posted all their tweets from beyond the coastal counties during the post-evacuation period. Thus, 37 users (2.7%) were classified as not evacuated, since they tweeted from both within and beyond the coastal counties during our post-evacuation time period.

**Table 2:** Evacuation rates and estimated evacuees by conglomerate.

Conglomerate	Users	Evacuated	% Evacuated	Pop. 2015	Estimated evacuees
Southern	105	81	77.1%	245,144	189,111
Central	560	280	50.0%	744,526	372,263
Northern	719	386	53.7%	370,497	198,904
Total	1,384	747	54.0%	1,360,167	760,278

For the entire study area, 54.0% (747 out of 1,384) of the local users evacuated, a finding consistent with previous evacuation studies. For example, the South Carolina hurricane evacuation behavioral study, Cutter et al. [59] indicated that 76.6% of respondents would evacuate for a major hurricane while only 21% expressed their intention of evacuating for a weaker hurricane. Our results from an actual evacuation fall in a middle position between the two intentions based on storm category. Although Matthew finally affected the state as a Category 1 hurricane, at the time the evacuation order was given the forecasted intensity was as a Category 3 (Fig 6). This may explain the higher evacuation compliance observed in our study in comparison with Cutter et al. [59], as the decision of evacuating is made upon forecasts and not storm intensity at landfall. In a fairly similar hurricane in terms of intensity, Hurricane Floyd in 1999, Dow and Cutter [9] reported an actual evacuation rate of 65%, which also supports our evacuation estimation. Another interesting finding that also lines up with Cutter et al. [59] is the noticeable geographic variation among the three conglomerates. The Southern Conglomerate experienced the highest evacuation rate (77.1%), followed by Northern Conglomerate (53.7%) and Central Conglomerate (50.0%). The South Carolina behavioral study [59] also identified more willingness to leave in the Southern Conglomerate (85.7% in major. hurricanes and 32% in weaker hurricanes) than in the Central Conglomerate (74.2%-18.0%) and Northern Conglomerate (70.7%-12.0%). With nearly 1.36 million people living along South Carolina’s coast in 2015, we estimated that 760,000 people evacuated in response to Hurricane Matthew. Based on South Carolina daily traffic counts, approximately 355,000 vehicles left the coastal counties during the period October 4-7^th^ [60]. As reviewed previously, most traffic-oriented studies only estimate the number of vehicles or the number of vehicles per household taken, not the number of people in each car [55, 59]. While the average household size in South Carolina coastal counties ranges from 2.42 to 2.84, many of these households live outside of mandatory evacuation zones. Rather than take this aggregate figure of household size as our occupancy per vehicle during the evacuation, we assumed a more conservative average occupancy of 2.0 people per car estimating that 710,000 people likely evacuated from the coastal counties—a number consistent with our estimates based on Twitter.

### 6.2 Where did residents travel to?

**Fig 9:**
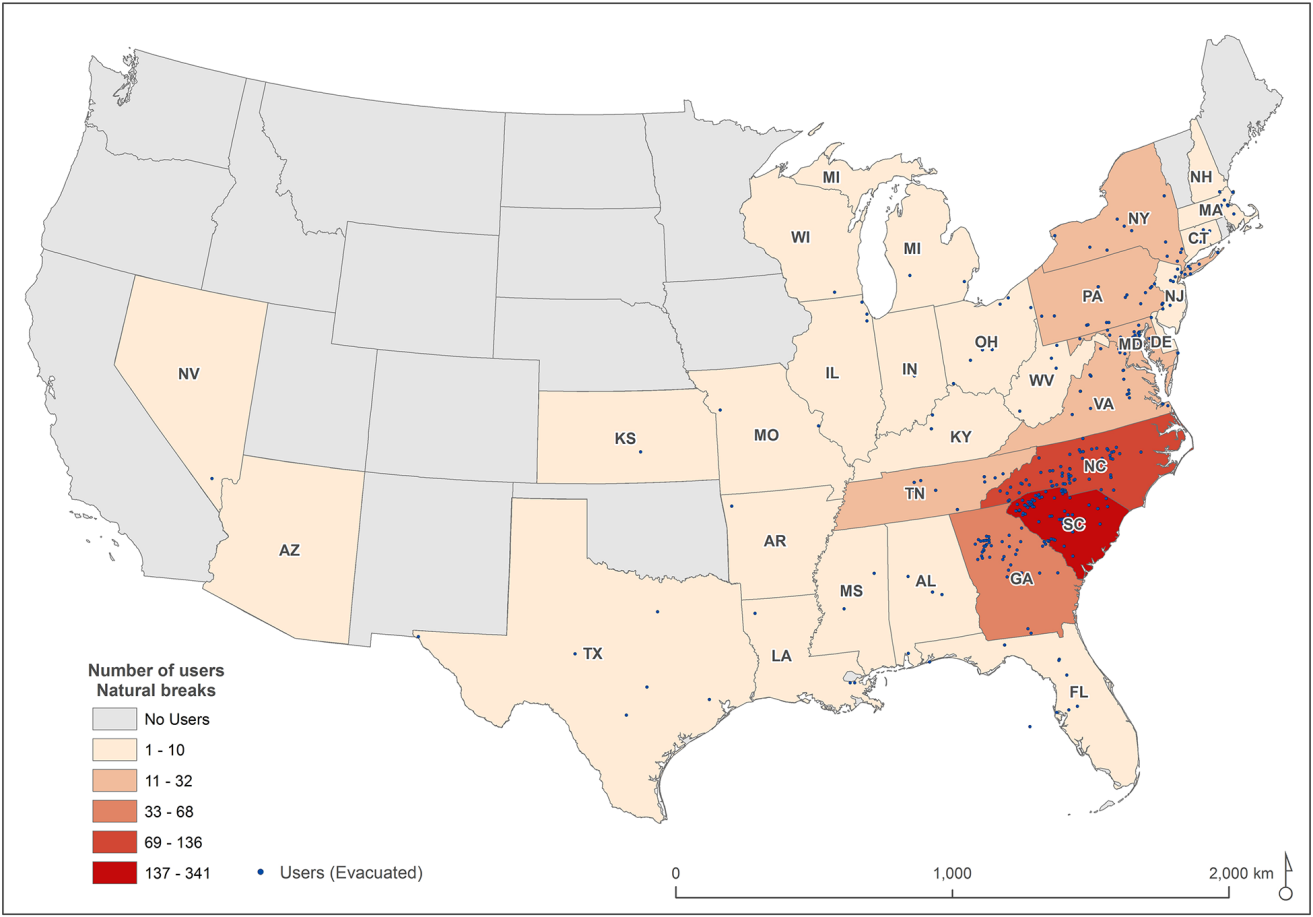
Evacuation destinations from South Carolina during Hurricane Matthew.

Most of the Twitter users were concentrated near the population centers of Charleston and Myrtle Beach. To visually represent destinations of the evacuees, locations were aggregated to the state scale (Fig 9). The majority of evacuated Twitter users (45.6%) did not leave South Carolina (Table 3). For those who moved out of state, 18.3% traveled to North Carolina and 9.1% to Georgia in areas far away from the coast. This confirms people’s propensity to evacuate to places relatively close to their homes, based on proximity to family and friends and the availability of hotels [59]. Also worth mentioning is the significant percentage of population evacuating to northeastern states (15.0%) such as Virginia, Pennsylvania, Maryland, New Jersey, and New York. In the study conducted by Cutter et al. [59], up to 70% residents reported that their potential evacuation shelter options were a friend’s or relative’s home (40.7%) or a hotel/motel (29.9%). The presence of family/friends, a larger accommodation capacity, and a good transportation corridor (I-95) may account for the northeastern destination preferences.

**Table 3:** Evacuation destinations from South Carolina during Hurricane Matthew.

State	Users	%
South Carolina	341	45.6%
North Carolina	136	18.2%
Georgia	68	9.1%
Virginia	32	4.3%
Pennsylvania	26	3.5%
Maryland	24	3.2%
New York	20	2.7%
Tennessee	19	2.5%
New Jersey	10	1.3%
Other states	71	9.5%

Surveys of South Carolina residents who evacuated from hurricanes Bertha and Fran in 1996 revealed that 15% and 28%, respectively, evacuated out-of-state [61, 62]. During Hurricane Floyd, 56% of South Carolinians who evacuated left the state [16]. This last figure matches closely with the 54.4% registered in our assessment, although it is considerably higher than the 35% reported by Cutter et al. [59] for a hypothetical evacuation.

### 6.3 When did evacuees return?

To investigate the return behavior, we tracked the locations of each tweet from the 747 evacuated users on a daily basis from October 9^th^ to October 19^th^ (Return dataset, Table 1). If the user’s Tweet location was within the eight coastal counties during this time period, then that user was counted as returned evacuee. People, against official recommendation, started to return home on October 9^th^ (11.0% of evacuated users returned) (Table 4). The following four days (October 10^th^– 13^th^) experienced the highest returns rates (18.6%, 14.9%, 15.1%, and 11.9%, respectively). October 14^th^ registered a noticeable drop in the return rate, which gradually slowed down from 4.8% to 2.3% on October, 10^th^. As of October 19^th^, 88.0% of the users had returned while 4.0% remained away from their home locations. The remaining 8.0 percent had not posted any messages during the 11-day period, therefore making it impossible to identify their locations.

**Table 4:** Return after evacuation.

Date	Users	% Returned	% Total
Sun. 10/09 from 10am	82	11.0%	11.0%
Mon. 10/10	139	18.6%	29.6%
Tue. 10/11	111	14.9%	44.4%
Wed. 10/12	113	15.1%	59.6%
Thu. 10/13	89	11.9%	71.5%
Fri. 10/14	36	4.8%	76.3%
Sat. 10/15	27	3.6%	79.9%
Sun. 10/16	19	2.5%	82.5%
Mon. 10/17	18	2.4%	84.9%
Tue. 10/18	17	2.3%	87.1%
Mon. 10/19	6	0.8%	88.0%
Not tweeted 10/09-10/19	60	8.0%	
Not returned	30	4.0%	
TOTAL	747	100.0%	

There are no prior studies of a timeline on the re-entry for South Carolina so we are unable to compare our results to previous hurricane experiences in the state. It is also possible that the data underestimate the return date for some evacuees simply based on no Twitter posts from individuals. This is an avenue for further research.

## 7 Limitations

There are a number of limitations to the approach used in this paper. First and foremost is the representativeness of Twitter data, which may not reflect the characteristics of the population under examination in terms of gender, race/ethnicity, socio-economic status, or age. While the emergent literature provides several attempts to understand the demographic profile of Twitter users [63,64], samples of Twitter users do have urban, gender (male), and race (Caucasian) biases. In other words, rural areas are less represented, as are females, and non-white users. However, the representativeness shortcoming is also shared with questionnaire surveys where respondents are often older, more educated, and include fewer minority respondents than the demographics of the area would suggest. The main difference between the two samples is that in the latter the researcher knows the biases (based on self-reports of respondents) and weights the results accordingly. The lack of any personal information about Twitter use (other than location) precludes knowing the representativeness of the sample.

Another limitation regarding the representativeness of the sample is the long-tail effect of the social media contributions where most of the social media contents (e.g. tweets) are contributed by a few users [65]. One implication of this effect is that active users can represent large numbers of individuals. For example, a single Twitter user who posts 25 tweets a day may weigh the same as 25 Twitter users who post once a day. In this sense, the spatiotemporal patterns of Twitter activity identified in the paper do not represent the total population, only accounting for the reaction of social media. This representation issue also applies to the evacuation analysis because the active users have a higher chance to be selected as the evacuation sample (1384 users) based on the steps described in Section 6. More research is needed to improve the capacity to infer the representativeness of Twitter samples regarding both demography and total population.

A third limitation is the selection bias of the available social media data, as people select to be included or to share their data, rather than statistically sampled. For example, Twitter users must grant consent to offer the geolocation of their tweets, so the locational information (origin-destination) could be highly skewed. It is unclear, for example, exactly what percentage of total Tweets contain geotags and how this might vary geographically during an emergency or disaster situation. In addition, since the Twitter streaming API only provides a small portion of all posted tweets, it is unclear how the streamed geotagged tweets were sampled from the tweets population.

Lastly, our approach is unable to systematically ascribe specific factors or motivations for the decision making to evacuate including the destination choice. The evacuation decision making during a hurricane is quite complex, requiring to consider different dimensions of evacuee behavior, such as the route choice, evacuation mode, choice of safe destination or mobilization time. There are extensive existing studies along this line and particularly notable are the work by Sadri et al. [66–69] and Hasan et al. [70,71]. Since this paper is not about evacuation decision making, but merely documenting the overt behavior (departure time, re-entry time, and location) as evidenced by social media data (tweets), the discussions of the underlying motivations of the route choice, choice of destination, and return time are beyond the scope this paper. The scope is also constrained by the limited information we can retrieve from the tweets comparing to traditional questionnaire surveys. However, since social media data contain social network information (e.g. followers, friends), we see a potential research avenue of utilizing social media data for understanding evacuation decision making, complementing the existing approaches of using questionnaire surveys or interviews [72,73].

## 8 Conclusions

This article examined Twitter reaction both spatially and temporally in response to a hurricane as a potential innovative approach for assessing protective action behavior and evacuation compliance in a timelier and cost-efficient manner. Considering that nearly half of the state-level emergency management agencies in the United States intend to leverage social media and open source information for public and situational awareness [74] this research confirms the utility of social media in monitoring public awareness and evacuation behavior in response to Hurricane Matthew.

Conducting spatiotemporal analyses permits the examination of public behavior as the hurricane moved northward. It also allows us to gauge the differential response by state emergency managers to the perceived threat of the hurricane based on their preparedness activities and calls for mandatory evacuations. Only Governors of the affected states can issue mandatory evacuation orders and the timing and extent of such emergency actions varies considerably even among neighboring states. In this sense, we observed a different temporal pattern in Twitter response during a tropical cyclone to the ones previously identified in the literature, as the most active period was recorded in advance to the advent of the storm and was linked to actions in the preparedness stage and distance from the storm track. A more detailed analysis of South Carolina evacuees using pre-evacuation and post-evacuation Twitter geotagged data enabled an estimation of the compliance rate for the mandatory evacuation, the timing of evacuee departure and return, and the destination of evacuees seeking shelter from the storm.

Our approach offers a solution to tackle several of the drawbacks of traditional assessments of evacuation rates through questionnaire surveys. These are frequently time-consuming and costly, and the response rates are often far from ideal. This alternative is cost-efficient and timely as the data can be collected in real-time, providing a remarkable sample size with successful results, as it has been shown. While the approach does have its limitations as mentioned previously, we do have a more robust measure of when Twitter users likely left, returned, and where they went (e.g. destination county). So the trade-off is the immediacy and relative accuracy of the timing of the evacuation and the destination of the evacuees versus the detailed motivation behind such behavioral responses assessed months later.

We believe the advantages outweigh the shortcomings of this approach to monitoring evacuation behavior. It provides complementary and near-real time data for assessing evacuation responses, and can be very useful when examined in tandem with traditional evacuation behavior methods. Future work should test the suitability of this approach in other emergency situations, as well as investigating the representativeness of Twitter samples for evacuation studies. Another line of research may take advantage of other sources of social media data. In this sense, it is worth noting that the pool of data available is considerably larger than what was used in this study (consider Facebook -79% of online adults-, Instagram-32%-, Pinterest-31%- in comparison with Twitter -24%-, [75]).A third line of research is to analyze the network properties and tweeting activities [76] of the 1384 Twitter users to identify the potential social factors that influence their decision to evacuate or stay, with an aim to predicting what kind of users are likely to evacuate via social network amplification [33]. Finally, research on the integration of new and traditional approaches and data sources is needed to explore avenues for better understanding evacuation decision making and improving evacuation management.

## Supporting information

S1 File Evacuation analysis related data and results. Including user locations during pre-evacuation period and post-evacuation period.
(XLSX)

S2 File Aggregated tweets and users for spatial analysis in the county level. (XLSX)

S3 File Aggregated number of Matthew-related tweets for temporal analysis. (XLSX)

S4 File Aggregated number of evacuation-related tweets for temporal analysis (XLSX)
